# Isoforms of Retinol binding protein 4 (RBP4) are increased in chronic diseases of the kidney but not of the liver

**DOI:** 10.1186/1476-511X-7-29

**Published:** 2008-08-27

**Authors:** Simone K Frey, Britta Nagl, Andrea Henze, Jens Raila, Beate Schlosser, Thomas Berg, Martin Tepel, Walter Zidek, Martin O Weickert, Andreas FH Pfeiffer, Florian J Schweigert

**Affiliations:** 1 Institute of Nutritional Science, University of Potsdam, Potsdam-Rehbrücke, Germany; 2 Medical Department, Devision of Hepatology and Gastroenterolgy, Campus Virchow-Clinic, Charité, University Medicine, Berlin; 3 Department of Medicine IV, Charité Campus Benjamin Franklin, Berlin, Germany; 4 Department of Clinical Nutrition, German Institute of Human Nutrition, Potsdam-Rehbrücke, Germany; 5 Department of Endocrinology, Diabetes and Nutrition, Charité University Medicine, Berlin, Germany

## Abstract

**Background:**

The levels of retinol-binding protein 4 (RBP4) – the carrier protein for Vitamin A in plasma – are tightly regulated under healthy circumstances. The kidney, the main site of RBP4 catabolism, contributes to an elevation of RBP4 levels during chronic kidney disease (CKD) whereas during chronic liver disease (CLD) RBP4 levels decrease. Little is known about RBP4 isoforms including apo-RBP4, holo-RBP4 as well as RBP4 truncated at the C-terminus (RBP4-L and RBP4-LL) except that RBP4 isoforms have been reported to be increased in hemodialysis patients. Since it is not known whether CLD influence RBP4 isoforms, we investigated RBP4 levels, apo- and holo-RBP4 as well as RBP4-L and RBP4-LL in plasma of 36 patients suffering from CKD, in 55 CLD patients and in 50 control subjects. RBP4 was determined by ELISA and apo- and holo-RBP4 by native polyacrylamide gel electrophoresis (PAGE). RBP4-L and RBP4-LL were analyzed after immunoprecipitation by mass spectrometry (MALDI-TOF-MS).

**Results:**

RBP4 isoforms and levels were highly increased in CKD patients compared to controls (P < 0.05) whereas in CLD patients RBP4 isoforms were not different from controls. In addition, in hepatic dysfunction RBP4 levels were decreased whereas the amount of isoforms was not affected.

**Conclusion:**

The occurrence of RBP4 isoforms is not influenced by liver function but seems to be strongly related to kidney function and may therefore be important in investigating kidney function and related disorders.

## Background

Retinol–binding protein 4 (RBP4) is a 21 kDa plasma protein, which is mainly secreted from the liver and adipose tissue and is known to transport retinol (ROH) in the blood. The binding of ROH to RBP4 guarantees the homeostatic regulation of plasma ROH levels, which are an essential aspect for a variety of physiological processes [1-3]. Recently, RBP4 levels have been reported to be elevated in insulin resistant subjects as well as in subjects with obesity and type 2 diabetes (T2DM) [4]. These diseases involve liver and kidney disorders in late stages [5,6].

In healthy individuals RBP4 is mainly synthesized in the liver and secreted into the circulation in a 1:1:1 complex with ROH (holo-RBP4) and transthyretin (TTR) [7,8]. The binding with TTR increases the molecular weight of RBP4 and thus prevents its glomerular filtration and catabolism in the kidney [9-13]. After releasing ROH into the target cells the remaining apo-RBP4 (unbound ROH) is rapidly filtered through the glomeruli and subsequently reabsorbed in the proximal tubular cells via the megalin-cubulin receptor complex and catabolized [1,14,15]. Importantly, dysfunctions of both, the liver and kidneys, are known to influence RBP4 homeostasis [13,16-18]: chronic kidney diseases (CKD) and chronic liver diseases (CLD) interfere with RBP4 metabolism through their action on RBP4 synthesis and catabolism [13,19].

RBP4 has been reported to occur in different isoforms in serum, namely holo-RBP4 (RBP4 bound to ROH) and apo-RBP4, which remains after the release of ROH into the target cell. In addition, little is known about RBP4 isoforms resulting from the truncation of RBP4: RBP4-L, which is truncated at one C-terminal leucine molecule (Leu-183), and RBP4-LL, which is truncated at a second leucine molecule (Leu-182 and Leu-183). The relative amounts of apo-RBP4 are increased in rats during acute renal failure and RBP4-L and RBP4-LL have been shown to be increased in hemo-dialysis patients [17,20,21]. It is assumed that renal dysfunction is closely linked to an increased occurrence of apo-RBP4 as well as RBP4-L and RBP4-LL in serum. However, sufficient data in these patients are lacking. In addition, it is unknown whether the liver, as site of RBP4 synthesis, may also contribute to the occurrence of RBP4 isoforms [22,23]. Thus, we examined RBP4 levels and isoforms in plasma of patients suffering from various CLD, as well as in patients with CKD, and compared the results with those obtained from healthy controls.

## Results

### Anthropometric and clinical parameters

Anthropometric and clinical characteristics of controls, CLD patients and CKD patients are shown in Table 1. There were no differences in age and BMI. Serum C-reactive protein (CRP) levels were higher in CLD and CKD patients compared to controls (P < 0.001, both), and CRP was elevated in CKD patients compared to CLD (P < 0.001). Serum creatinine, a parameter of kidney function, was elevated in CKD compared to controls and CLD (P < 0.001, both).

**Table 1:** Clinical and biochemical characteristics of controls, patients with CLD and CKD.

	**Controls**	**CLD**	**CKD**
N (m/f)	50 (27/23)	55 (30/25)	36 (26/10)
Age (years)	55.0 (48.2 – 60.0)	50.0 (44.0 – 58.0)	59.0 (45.3 – 68.8)
BMI (kg/m^2^)	24.4 (22.5 – 26.2)	24.0 (21.3 – 25.9)	25.3 (22.2 – 27.7)
Glucose (mmoL/L)	4.73 (4.45 – 5.17)	5.27 (4.82 – 5.94) *	5.24 (4.63 – 5.94) *
CRP (nmoL/L)	0.0 (0.0 – 0.0)	43.5 (39.3 – 52.1) *	342.9 (104.8 – 800.0) * ^#^
Creatinine (μmoL/L)	68.8 (63.5 – 75.5)	63.3 (55.7 – 73.9) *	239.4 (180.0 – 528.4) * ^#^
Protein (g/L)	66.5 (63.5 – 70.2)	50.7 (45.2 – 57.1) *	65.0 (59.0 – 70.0) ^#^
AST [μkat/L]	0.35 (0.28 – 0.40)	0.89 (0.36 – 1.51) *	0.30 (0.24 – 0.43)^#^
ALT [μkat/L]	0.17 (0.12 – 0.24)	0.82 (0.55 – 1.39) *	0.37 (0.21 – 0.52) * ^#^
ALP [μkat/L]	1.06 (0.79 – 1.31)	1.73 (0.99 – 2.48) *	1.32 (0.97 – 1.62) *
GGT [μkat/L]	0.30 (0.21 – 0.43)	1.03 (0.44 – 2.21) *	0.53 (0.32 – 1.41) *
Haemotocrit	0.41 (0.38 – 0.44)	0.42 (0.38 – 0.46)	0.32 (0.29 – 0.37) * ^#^
Haemoglobin (g/L)	138.5 (130.0 – 149.8)	145.0 (126.8 – 155.3)	107.5 (97.5 – 125.0) * ^#^

Data are expressed as median with 25th and 75th percentiles. BMI, body mass index; CRP, C-reactive protein; AST, aspartate aminotransferase; ALT, alanine aminotransferase; ALP, alkaline phosphatase; GGT, gamma glutamyl transferase.

* = significantly different from controls (p < 0.05). # = significantly different from CLD (p < 0.05).

Standard tests of liver function such as alanine aminotransferase (ALT), gamma-glutamyl transferase (GGT), alkaline phosphatase (ALP) and aspartate aminotransferase (AST) concentrations showed increased levels in the CLD group compared to the values in the controls (P < 0.001). The levels of ALT, GGT and ALP were also increased in the CKD group compared to controls (P < 0.01). However, AST and ALT were markedly lower in the CKD compared to the CLD group (P < 0.001).

### Biochemical variables of the RBP4-complex

Compared to controls, RBP4 levels were lower in CLD (P < 0.001), but highly elevated in patients with CKD (P < 0.001, Table 2). Among CLD patients those with c2-cirrhosis (ethanol-induced) showed lowest RBP4 values compared to CLD patients with fibrosis or hepatic cancer (P < 0.001, Table 3). Serum ROH levels were increased in CKD patients compared to CLD patients (P < 0.001) and in controls compared to CLD (P < 0.001). In patients with fibrosis, ROH levels were elevated compared to CLD patients with HCC and c2-cirrhosis (P < 0.01). The highest TTR levels were observed in controls compared to CLD and CKD (P < 0.001, Table 2).

**Table 2:** Biochemical variables of the ROH-RBP4-complex in plasma of controls, patients with CLD and CKD.

	**Controls**	**CLD**	**CKD**
RBP4 (μmoL/L)	2.17 (1.78 – 2.52)	0.97 (0.61 – 1.27) *	3.75 (2.63 – 5.21) * ^#^
ROH (μmoL/L)	1.46 (1.29 – 1.66)	0.94 (0.62 – 1.22) *	1.62 (1.03 – 2.69)^#^
TTR (μmoL/L)	6.14 (5.22 – 7.01)	1.16 (0.91 – 1.83) *	4.22 (2.66 – 6.02) * ^#^
RBP4/ROH ratio^1^	1.39 (1.15 – 1.78)	1.04 (0.90 – 1.32) *	1.88 (1.51 – 3.04) * ^#^
RBP4/TTR ratio ^2^	0.36 (0.30 – 0.41)	0.67 (0.41 – 1.13) *	0.76 (0.61 – 1.49) *
Holo-RBP4 (%)	86.4 (80.7 – 88.8)	85.0 (77.9 – 93.4)	67.0 (54.0 – 76.3) *
Apo-RBP4 (%)	13.6 (11.2 – 19.3)	15.0 (6.3 – 22.0)	32.5 (23.8 – 42.0) *
RBP4-L (%)^3^	45.0 (24.5 – 73.0)	33.0 (17.5 – 48.0) *	86.5 (39.0 – 143.0) *
RBP4-LL (%)^3^	0.0 (0.0 – 5.5)	0.0 (0.0 – 6.0)	18.0 (5.75 – 55.8) *

Data are expressed as median with 25th and 75th percentiles. RBP4, retinol binding protein 4; ROH, retinol; TTR, transthyretin.

* = significantly different from controls (p < 0.05). # = significantly different from CLD (p < 0.05).

^1 ^The RBP4/ROH ratio is the molar ratio of serum RBP4 to serum ROH.

^2 ^The RBP4/TTR ratio is the molar ratio of serum RBP4 to serum TTR.

^3 ^The intensity of the non-truncated RBP4 (nRBP4) was set 100% and intensities of RBP4-L and RBP4-LL were expressed in % of nRBP4.

**Table 3:** Biochemical variables of the ROH-RBP4-TTR complex in plasma of CLD patients classified for individual liver diseases.

	**Fibrosis^1 ^(n = 38)**	**HCC^2 ^(n = 10)**	**Cirrhosis (n = 7)**
ROH (μmoL/L)	1.15 (0.87 – 1.40)	0.68 (0.55 – 0.85) *	0.51 (0.38 – 0.72) *
RBP4 (μmoL/L)	0.98 (0.64 – 1.36)	1.02 (0.76 – 1.02)	0.53 (0.41 – 0.82) *
TTR (μmoL/L)	1.44 (1.86 – 3.09)	1.02 (0.91 – 1.26)	1.00 (0.93 – 1.40)
RBP4/ROH ratio ^3^	0.97 (0.83 – 2.17)	1.53 (1.00 – 2.13)	1.07 (0.97 – 1.26) *
RBP4/TTR ratio ^4^	0.71(0.38 – 1.13)	1.05 (0.55 – 1.36)	0.51 (0.41 – 0.61) ^#^
RBP4-L (%)^5^	33.00 (17.00 – 46.00)	31.00 (16.50 – 56.00)	44.00 (20.00 – 58.00)
RBP4-LL (%) ^5^	0.00 (0.00 – 6.00)	6.00 (0.00 – 8.00)	0.0 (0.0 – 0.0)

Data are expressed as median with 25th and 75th percentiles.

^1 ^Fibrosis stage 0–4, ^2 ^HCC = Hepato-cellular carcinoma;

^3 ^The RBP4/ROH ratio is the molar ratio of serum RBP4 to serum ROH.

^4 ^The RBP4/TTR ratio is the molar ratio of serum RBP4 to serum TTR.

^5 ^The intensity of the non-truncated RBP4 (nRBP4) was set 100% and intensities of RBP4-L and RBP4-LL were expressed in % of nRBP4.

* = significantly different from Fibrosis patients < (p < 0.05). # = significantly different from HCC patients (p < 0.05).

The molar ratio of RBP4 to ROH was significantly decreased in the CLD group compared to controls (P < 0.001) as well as to CKD (P < 0.001). The CKD group showed the highest value of the RBP4 to ROH ratio compared to controls as well as to the CLD group (P < 0.001). An excess of RBP4 over ROH indicates an elevation in free RBP4 and thus apo-RBP4 (unbound ROH). This is supported by the significant correlation of apo-RBP4 and the RBP4-ROH ratio (Spearman Rho r = 0.565, P < 0.01). The molar ratio of RBP4 to TTR was increased in CLD and CKD compared to controls (P < 0.001, both, Table 2).

### Relative amounts of apo- and holo-RBP4

**Figure 1:**
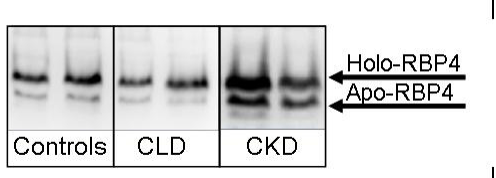
**Representative polyacrylamide gel electrophoresis-immunoblotting of apo- and holo-RBP4 bands in serum of controls, patients with chronic liver disease (CLD) and chronic kidney disease (CKD)**. Relative amounts were calculated by comparing the intensity of the apo-band to the holo-RBP4 bands of each lane and are displayed as percentage of total intensity per lane.

Analysis of band area after non-denaturating PAGE immunoblotting was used to calculate the relative amount of apo- and holo-RBP4. The relative amount of holo-RBP4 was higher in plasma of controls as well as in CLD compared to CKD (P < 0.001, both). Conversely, apo-RBP4 was detected in higher quantities in CKD compared to controls and CLD patients (P < 0.001, Figure 1).

### Relative amounts of RBP4-L and RBP4-LL (by MALDI-TOF-MS)

**Figure 2:**
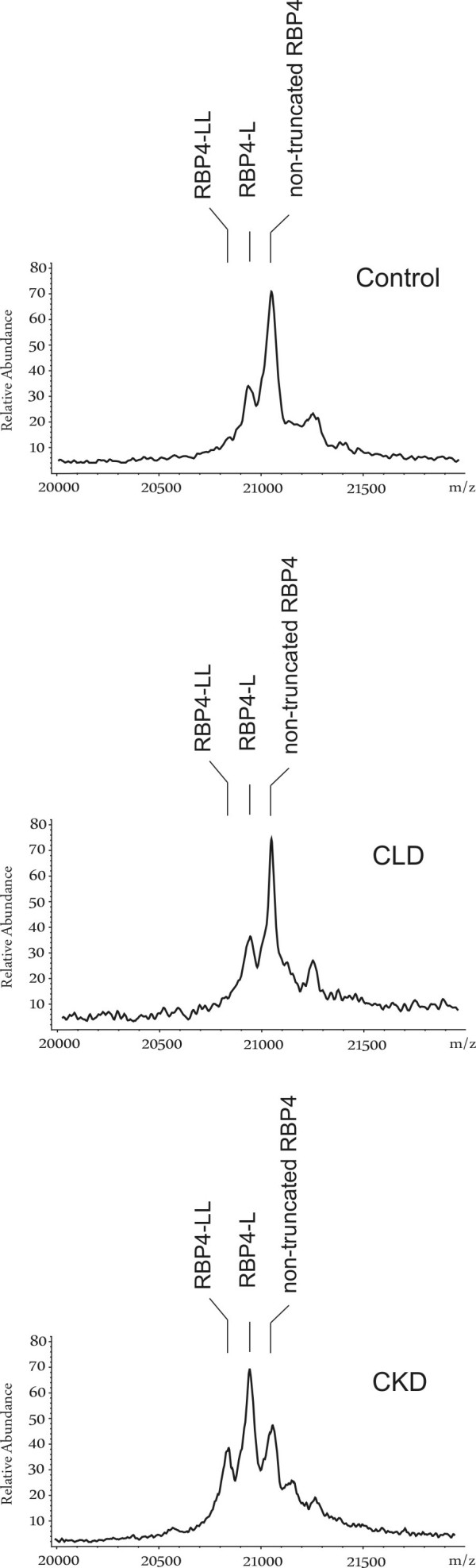
**Representative MALDI spectra of RBP4 in a healthy control, a chronic liver disease (CLD) patient and a chronic kidney disease (CKD) patient.** Control and CLD patient show the non-truncated RBP4 (1 = 21.065 Da) and the RBP4-L peak (2 = 20.950 Da) whereas the RBP4-LL peak (3 = 20.837 Da) is solely present in the CKD patient.

**Figure 3:**
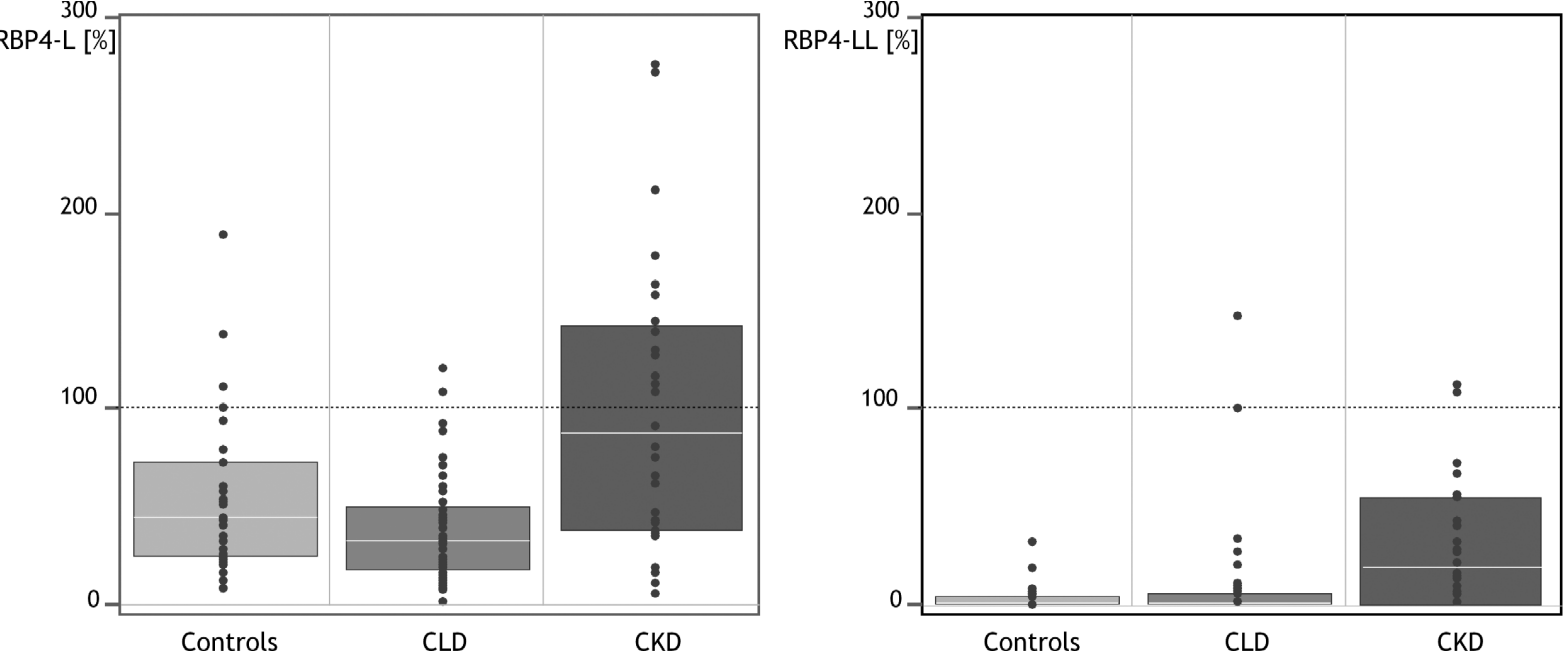
**Relative amounts of RBP4-L and RBP4-LL in controls, patients with chronic liver disease (CLD) and chronic kidney disease (CKD).** The intensities of RBP4-L and RBP4-LL in the sera of the CLD group, the CKD and the control group were calculated in relation to the peak height of the non-truncated RBP4 peak (21.065 Da), which was set 100%. The peak heights of RBP4-L and RBP4-LL are expressed as percentage of the non-truncated RBP4. All peak heights were determined in a "valley-to-valley" procedure. Boxes represent interquartile range with median (white bar); black dots represent single values of each subject.

RBP4 immunoprecipitation and subsequent MALDI-TOF-MS analysis was used to detect RBP4-L and RBP4-LL (Figure 2). In controls, non-truncated RBP4 (nRBP4) was the most abundant form and was set to 100%. RBP4-L and RBP4-LL were analysed in a "valley-to-valley" procedure and expressed as per cent of nRBP4. RBP4-L occurred in relative amounts of nRBP4 with a median of 45% and RBP4-LL with 0%. In CKD patients both, RBP4-L (87%) and RBP4-LL (18%), were significantly elevated compared to CLD and controls (P < 0.001, both, Table 2 and Figure 3).

### Correlations among RBP4 levels as well as RBP4 isoforms and parameters of liver and kidney function

With regards to liver function, plasma RBP4 and ROH levels were inversely correlated with AST (r = -0.659, r = -0.494), ALT (r = -0.510, r = -0.314), ALP (r = -0.187, r = -0.288) and GGT (r = -0.312, r = -0.203, respectively, P < 0.05, all). AST was correlated with holo-RBP4 (r = 0.330) and inversely with apo-RBP4 (r = 0.317, P < 0.05, both). In addition, AST levels were inversely correlated with RBP4-L (r = -0.421) and RBP4-LL (r = -0.297, P < 0.01, both). ALP was inversely correlated with RBP4-L (r = -0.248, P < 0.01).

With regards to kidney function, there was a correlation between serum creatinine and RBP4 levels (r = 0.633), apo-RBP4 (r = 0.674), RBP4-L (r = 0.494) and RBP4-LL (r = 0.438) as well as ROH (r = 0.396, P < 0.01, all). Holo-RBP4, in contrast, was inversely correlated with serum creatinine (r = -0.678, P < 0.01). In CLD, serum creatinine was correlated with RBP4 (r = 0.535), RBP4-L (r = 0.421, P < 0.01, both) and ROH levels (r = 0.381, P < 0.05).

## Discussion

This study was designed to investigate the effect of CLD and CKD on RBP4 isoforms and to identify factors influencing and/or generating RBP4 isoforms. We were able to show that the relative amount of RBP4 isoforms (apo-RBP4, RBP4-L, RBP4-LL) was increased in CKD patients, but not in CLD patients, in comparison to controls. Our results also show that RBP4 levels were significantly elevated in serum of CKD patients compared to both, CLD patients and controls. In contrast, RBP4, TTR and ROH levels were significantly decreased in CLD patients, as compared to CKD patients and controls.

*Jaconi et al. *[20] investigated RBP4-L and RBP4-LL in the serum of hemo-dialysis patients and regarded the occurrence of RBP4 isoforms to be specific for CKD [11,17]. To date, RBP4 isoforms have been investigated exclusively in a small number of patients ([1] and [10], respectively) suffering from CKD [11,17] and not in CLD patients. Our data show that RBP4-L and RBP4-LL, which are truncated at the C-terminal end of the molecule, were increased in CKD (Figure 1). In contrast to that, in CLD patients – irrespective of the kind of liver disease – there were no increased amounts of RBP4-L and RBP4-LL, thus supporting the relation between RBP4 isoforms and kidney function. The increased survival and retention of RBP4 in the circulation during CKD may contribute to the increased truncation of RBP4. Although there is evidence that a specific carboxypeptidase is responsible for the truncation [17,20], the physiological impact of RBP4-L and RBP4-LL is not known. However, RBP4-L and RBP4-LL isolated from CKD serum, inhibit chemotaxis and oxidative metabolism of polymorphonuclear leucocytes. These alterations in leucocytes activity may disturb immune defense in these patients [24]. In addition, the C-terminal end of RBP4 is involved in ROH binding, and therefore, RBP4 modifications may also influence the interaction with TTR [7,25].

Additionally, we confirmed that RBP4, TTR and ROH levels in various liver diseases were markedly depressed, particularly in patients with c2-cirrhosis or hepato-cellular carcinoma, which is in accordance with results of previously published studies [16,26-28]. This decrease is due to a loss of functional hepatic tissue resulting in decreased synthesis of RBP4 and TTR and decreased release of the ROH-transport complex into the circulation [23,27].

In patients with CKD, the levels of RBP4 were markedly elevated and therefore the molar ratio of RBP4 to TTR was increased. In healthy states, TTR is present in a 3–5 fold molar excess in plasma and the serum RBP4/TTR ratio is approximately 0.4 whereas in CKD patients an increase in the RBP4/TTR molar ratio up to 1.06 has been reported [16,18,29,30]. This is consistent with the 3-fold elevated RBP4/TTR ratio from 0.36 in controls to 0.96 in CKD in our study. Due to the increase of RBP4 and the simultaneous fall in TTR levels in CKD, almost one molecule of TTR and one molecule of RBP4 are present in the circulation [16,18,31]. The decrease in TTR levels in CKD may be due to malnutrition and/or infectious disease [16,29].

The kidneys play an important role in the recycling of RBP4 since RBP4 catabolism is disturbed in CKD patients [16,31]. According to previous studies elevated serum creatinine levels, a marker for kidney dysfunction, are associated with high serum concentrations of RBP4 [16,32]. This is due to the loss of functional tissue and/or the entire nephron in kidney failure, which leads to decreased filtration of creatinine and abnormal survival of small serum proteins resulting in an increase of their serum levels [10,33]. This might explain the increased RBP4 levels in CKD (Table 2). Under physiological conditions 98% of RBP4 is bound to ROH (holo-RBP4) and 2% circulate ROH free as apo-RBP4 [18,34]. In this study we show that the percentage of plasma apo-RBP4 is highly increased in CKD patients compared to controls and CLD patients, thus supporting early findings [20,35]. Nearly all of the apo-RBP4 is normally glomerularly filtered and reabsorbed by the kidney proximal convoluted tubules. The increase in the molar ratio of RBP4 to ROH in our CKD patients indicates an excess of RBP4 over ROH, leading to an increase in RBP4 unbound to ROH, which is consistent with the increase in apo-RBP4. The altered holo- to apo-RBP4-ratio in CKD complies also with previous results indicating that impaired kidney function compromises sufficient metabolisation of apo-RBP4 from serum [14,20,31,36]. This finding is confirmed by the correlation of apo-RBP4 and serum creatinine in our study.

The alterations in RBP4 metabolism during CKD are of interest in relation to T2DM since T2DM patients are exposed to increased oxidative stress which has been reported to be linked to endothelial dysfunction [37]. It is known that T2DM patients often suffer from kidney dysfunction [38] and therefore the RBP4-L and RBP4-LL may further enhance oxidative stress through their action on polymorphonuclear leucocytes [24].

## Conclusion

The results of the study show that the disturbed catabolism of RBP4 in CKD results in an increase in RBP4 isoforms including apo-RBP4, RBP4-L and RBP4-LL – whereas the generation of RBP4 isoforms is not influenced by liver function. However, both CKD and CLD do influence serum RBP4 levels. Since the increase in RBP4 isoforms was not observed in patients suffering from various CLD, the important physiological function of the kidneys in that context is emphasized and it may be suggested that impaired catabolism of RBP4 in the kidneys leads to an accumulation of RBP4 isoforms in serum. These results support the hypothesis that the C-terminal truncation of RBP4 may be specific during CKD.

## Methods

### Subjects

Sera of 50 healthy subjects were obtained from the Department of Clinical Nutrition, German Institute of Human Nutrition, Potsdam-Rehbrücke, Germany. The inclusion criteria for healthy subjects were no known diagnosis of any kidney, liver or metabolic disease, such as obesity/adiposity, diabetes or hypertension, and no drug intake.

Sera of 45 patients with CKD were obtained from the Department of Medicine IV, Charité Campus Benjamin Franklin, Berlin, Germany. Subjects were characterized according to their estimated glomerular filtration rate (eGFR) which was calculated according to the MDRD formula [40]. In the CKD group patients with moderately decreased (30 – 60 ml/min/1.73 m^2^) and severely decreased (< 30 ml/min/1.73 m^2^) eGFR were included [41].

Sera of 63 patients with CLD were obtained from the Department of Medicine IV, Charité Campus Virchow, Berlin, Germany. Of these patients, 10 were diagnosed with fibrosis METAVIR stage 0 – 1.5, 12 with fibrosis METAVIR stage 2 – 2.5, 9 with METAVIR stage 3, 7 with fibrosis METAVIR stage 4, 10 with hepato-cellular cancer and 7 with c2 cirrhosis. Cirrhosis diagnosis was made depending on histopathologic, clinical and laboratory findings. Staging was differentiated according to fibrosis: Stage 1 = zone 3 perisinusoidal/pericellular fibrosis, focal or diffuse; stage 2 = focal of diffuse periportal fibrosis together with zone 3 perisinusoidal/pericellular fibrosis; stage 3 = focal and diffuse bridging necrosis together with perisinusoidal/pericellular fibrosis and portal fibrosis; stage 4 = Cirrhosis. Body mass index (BMI) was calculated by the formula: weight (kg)/height(m^2^).

### Laboratory analyses

Blood samples were collected by the attending physician after an overnight fast. Serum was stored at -80°C until processing. The study protocol was approved by the Ethics Committees of the Universities of Charité Berlin and Potsdam. Informed consent was obtained from each subject. AST, ALT, GGT, ALP, total protein, albumin, serum creatinine, serum albumin, bilirubin, glucose levels were measured by routine laboratory methods.

### Determination of ROH, RBP4, TTR and CRP

For separation and quantification of ROH a gradient reversed-phase HPLC system was used as previously described [39]. Briefly, 200 μl of ethanol were added to 100 μl plasma (1:1 diluted with water). Afterwards, plasma was extracted twice with n-hexane, stabilized with 0.05% butylated hydroxyluene (BHT), vortexed and centrifuged for 10 min at 1500 *g*. The supernatants were removed and evaporated under nitrogen and reconstituted in 200 μl isopropanol and injected into the HPLC system (C30 carotenoid columm, 5 μm, 250 × 4,6 mm, in line with C18 pre-columm, solvent A methanol:water (90:10 v:v, with 0,4 g/l ammonium acetate in water), solvent B methanol:methyl-tert-butyl-ether:water (8:90:2 v:v:v, with 0,1 g/l ammonium acetate in water).

Serum levels of RBP4 and TTR were measured by ELISA using polyclonal rabbit anti-human antibodies for RBP4 and Prealbumin (Dako, Hamburg, Germany) as previously described [40,41]. Determination of CRP was performed by ABX Pentra CRP CP, a latex-enhanced immunoturbidimetric assay (ABX Diagnostics, Monpellier, France).

### Immunoprecipitation of RBP4 and subsequent analysis by MALDI-TOF-MS

For immunoprecipitation 10 μl of serum sample was incubated with an equal amount of Sephadex G 15 and 5 μl polyclonal rabbit anti-human RBP4 (Dako, Hamburg, Germany) at room temperature for 18 hours, centrifuged at 13,000 rpm for 20 min at room temperature. After removing the supernatant, the protein-antibody complex was washed twice with PBS and once with HEPES. The samples were then applied on the MALDI target using 2 μl of sample. Afterwards, 2 μl saturated sinapinic acid solution was placed on a serum drop and dried. The matrix solution contained 1 mg sinapinic acid and an equal amount of 1% trifluoroacetic acid and acetonitrile. MALDI mass spectra were obtained using a Reflex II MALDI-TOF mass spectrometer (Bruker-Daltronik, Bremen, Germany) which was performed in a linear mode at 20 k acceleration voltage. For ionisation, a nitrogen laser (337 nm, 3 ns pulse width, 3 Hz) was used. For optimisation of the mass spectra, the laser was aimed either at the central area of the sample or at the outmost edge of the crystal rim. All spectra were measured using external calibration. As the ionization efficiencies of non-truncated RBP4, RBP4-L and RBP4-LL are similar, the peaks in the mass spectra reflect the relative amounts of RBP4-L and RBP4-LL [17]. Therefore peaks were analysed "valley-to-valley" and are expressed as percentage of non-truncated RBP4 (nRBP4).

### Determination of relative amounts of apo- and holo-RBP4

Relative amounts of holo-RBP4 and apo-RBP4 in serum were assessed by using nondenaturating polyacrylamide gel electrophoresis (PAGE) with subsequent immunoblotting analysis. Under these conditions retinol remains bound to RBP4 and due to the higher molecular weight of holo-RBP4 (+ 286 Da), two bands may be detected. The PAGE was performed according to Siegenthaler and Saurat with slight modifications [17]. Briefly, the resolving gel was prepared using 12% acrylamide/bisacrylamide and 0.05% ammoniumpersulfate (APS) and 0.075% N,N,N',N'-tetramethylethylenediamine (TEMED) as crosslinker in 0.375 Tris/HCl, pH 8.8. The stacking gel (4% acrylamide/bisacrylamide, 0.05% APS, 0.1% TEMED) was prepared in 0.125 M Tris/HCl, pH 6.8. 10 μl of serum diluted 1:20 in sample buffer (0.125 Tris/HCl, 2.74 M glycerol, 0.1 mM bromphenol blue, pH 6.8) was applied to each slot, with 12 samples per gel. The electrophoresis conditions were 25 mA per gel for 30 to 45 min at room temperature. The proteins were separated according to their electrophoretic mobilities and subsequently transferred onto a polyvinyl difluoride (PVDF) sheet. Immunoreactive bands were visualized by using rabbit anti-human RBP4 (Dako) and peroxidase-coupled swine anti-rabbit immunoglobulins (Dako). Antibody binding was visualized using the Luminol reaction (BM Chemiluminescence Blotting Substrate, Roche Diagnostics, Mannheim, Germany). Since the binding of ROH persists under nondenaturating conditions two bands are obtained per lane, apo- and holo-RBP4. Band intensity of both RBP4 isoforms was read with an imager (Bio-Rad, Munich, Germany) and with the Quantity One^® ^software (Bio-Rad). The relative amounts of apo- and holo-RBP4 per lane are expressed as per cent of total intensity of each lane. However, since apo- and holo-RBP4 are the only visible bands, the sum of the relative quantities of both isoforms equals 100% per lane.

### Statistical procedures

Results are shown as medians and interquartile ranges. Statistic calculations were performed using SPSS 14.0 (SPSS statistical package, SPSS Inc., Chicago, USA). The Kruskal-Wallis test was used to test for significant differences in continuous variables between the groups. If there was a significant effect, Mann-Whitney U-rank test was performed to describe differences in proportions between cases and controls. Spearman rank correlation coefficients were used to test the association between laboratory parameters and variables of ROH-RBP4 transport complex. Values of P < 0.05 were regarded as significant.

## Abbreviations

CKD: Chronic kidney disease; CLD: Chronic liver disease; MALDI-TOF-MS: Matrix-assisted laser desorption ionization time-of-flight mass spectrometry; RBP4: Retinol-binding protein 4; T2DM: type 2 diabetes.

## Competing interests

The authors declare that they have no competing interests.

## Authors' contributions

SKF and BN carried out sample analysis and prepared the manuscript. MOW and AHFP recruited blood samples of the control group. TB, BS, MT and WZ recruited blood samples of patients. AH, JR reviewed the manuscript and FJS created the study design. All authors have read and approved the final version of the manuscript.
